# Choline Supplementation Does Not Promote Atherosclerosis in CETP-Expressing Male Apolipoprotein E Knockout Mice

**DOI:** 10.3390/nu14081651

**Published:** 2022-04-15

**Authors:** Heidi L. Collins, Steven J. Adelman, Dustie N. Butteiger, Jonathan D. Bortz

**Affiliations:** 1 VascularStrategies LLC, 5110 Campus Drive, Suite 137, Plymouth Meeting, PA 19462, USA; sadelman@vascularstrategy.com; 2 Human Nutrition and Health, Nutrition Science, Balchem Corporation, 52 Sunrise Park Road, New Hampton, NY 10958, USA; dbutteiger@balchem.com (D.N.B.); jbortz@balchem.com (J.D.B.)

**Keywords:** choline, atherosclerosis, trimethylamine N-oxide, gut microbiota, cardiovascular disease

## Abstract

Dietary trimethylamines, such as choline, metabolized by intestinal microbiota to trimethylamine are absorbed by the gut and oxidized to trimethylamine N-oxide (TMAO). The objective of this study was to determine the effect of choline supplementation on atherosclerosis progression in *Apoe^−/−^* mice expressing human cholesterol ester transfer protein (hCETP) using the same diets as in previously reported studies. Mice expressing hCETP, after transfection with AAV2/8-hCETP, were fed an 18% protein diet with either 0.09% (standard chow), 0.5% or 1% choline for 16 weeks. Control mice not transfected with hCETP were fed 1% choline. Dietary choline supplementation increased plasma TMAO levels at 8 and 16 weeks. When atherosclerotic lesions were measured in the thoracic aorta and aortic root, there were no differences between any of the treatment groups in the amount of plaque development at either site. Throughout the study, no significant changes in plasma lipids or major classes of lipoproteins were observed in hCETP-expressing mice. Plasma-oxidized low density lipoprotein, myeloperoxidase and high density lipoprotein inflammatory index were measured at 16 weeks, with no significant changes in any of these inflammatory markers between the four treatment groups. Despite increasing plasma TMAO levels, dietary choline supplementation in *Apoe^−/−^* mice expressing hCETP did not promote atherosclerosis.

## 1. Introduction

Over the last 60 years, the association of lipid abnormalities with cardiovascular disease (CVD) has assumed a bedrock status in the medical, scientific and even lay understanding of the pathogenesis underlying atherosclerosis. Atherosclerosis, one of the leading causes of CVD, has proved to be complex and can be affected by not only dyslipidemia but many other factors, such as smoking, diabetes, physical activity, nutrition, genetic background, inflammatory status, physical activity and hypertension.

The intestinal microbiome has received an increasing amount of attention as a contributor to the progression and/or increased risk of diseases such as obesity and type II diabetes, so it would not be surprising if the microbiome was found to be a contributor to CVD. An emerging theory suggests a pivotal role for the gastrointestinal microbiome in transforming carnitine and metabolites of phosphatidylcholine into TMA (trimethylamine). TMA is absorbed and then converted by hepatic flavin mono-oxidase 3 (FMO3) to trimethylamine oxide (TMAO), which is regarded as proatherogenic independent of dyslipidemia, environmental factors, diabetes and hypertension [1,2]. The fact that trimethylamine compounds are found in the highest concentrations in foods that have long been associated with cardiovascular risk (liver, eggs and red meat) would seem to support this hypothesis.

Choline, an essential nutrient, is found mainly in animal products but can also be found in plants, especially cruciferous vegetables and certain legumes [3]. Choline can also be produced via de novo synthesis in the liver; however, de novo synthesis of choline is not adequate to meet human requirements [4]. The recommended daily intake (RDI) of choline is 425 mg/day for women and 550 mg/day for men [4]. Once choline is made or consumed via dietary sources, it is converted to phosphatidylcholine (PC), which is one of the most abundant phospholipids found in mammalian cell membranes [5,6]. Choline serves as the major source of methyl groups through the synthesis of S-adenosylmethionine [7]. These methyl reactions are critical in the biosynthesis of lipids, maintenance of liver health [8], facilitation of metabolic pathways [6,9] and reduction in plasma homocysteine [10].

Previous studies supporting the metabolomic association of the choline metabolite, TMAO, with CVD used the *Apoe^−/−^* atherosclerosis mouse model fed either a normal rodent chow (0.09% total choline, *w*/*w*), an intermediate dose (0.5% total choline), a high-choline dose (1.0%) or TMAO (0.12%) at the time of weaning [1,2]. In these studies, researchers fed high amounts of choline to elevate plasma TMAO levels. It is worth noting that the lowest choline dose supplemented in the normal mouse chow corresponds to an equivalent human dose of 900 mg/day, and the highest dose supplemented is equivalent to a human dose of 10,000 mg/day (20X greater than the Recommended Daily Intake). In early studies, Wang [2] found increased TMAO plasma concentrations correlated to an increase in total aortic root atherosclerotic plaque area in mice supplemented with either increased choline or TMAO for 16 weeks. However, these results have not been consistently reproduced when supplementing choline in such high amounts [11,12,13].

Although the *Apoe^−/−^* mouse model is employed frequently, it is notable for the absence of a key protein involved in human reverse cholesterol transport: cholesterol ester transfer protein (CETP). Collins et al. used male *Apoe^−/−^* mice transfected with hCETP and supplement-fed L-carnitine on a Western diet and were not able to reproduce earlier findings of increased atherosclerosis with increased plasma TMAO concentrations [12]. In fact, they found that increased plasma TMAO was associated with reduced progression of aortic lesions. Therefore, the experiment performed by Wang and colleagues [2] was repeated in the present study using the same background diets and two dose levels of choline in male *Apoe^−/−^* mice with and without CETP expression on a normal diet. The data presented herein do not support a relationship between increased plasma TMAO and aortic lesion progression when supplementing with high doses of choline for 16 weeks.

## 2. Materials and Methods

### 2.1. Animals

Mouse study protocols were approved by the Institutional Animal Care and Use Committee of VascularStrategies and adhered to the criteria outlined in the ‘*Guide for the Care and Use of Laboratory Animals*’. Male *Apoe^−/−^* mice at age 6–7 weeks purchased from Jackson Laboratories (Bar Harbor, ME, USA) were transfected at 7–8 weeks with an adeno-associated viral vector containing the human CETP gene (AAV2/8-hCETP, purchased from ReGenX Biosciences, Rockville, MD, USA) as previously described by Tanigawa et al. [14]. Expression of CETP was confirmed 2 weeks after CETP-AAV transfection using a commercially available ELISA kit from Cell Biolabs, Inc. (San Diego, CA, USA). During acclimation and transfection, animals were maintained on Purina Lab Diet 5001. Two weeks post-transfection, mice were serpentine-sorted into groups based on total cholesterol levels and switched to a normal chow (TD2018) containing 0.09% *w/w* choline, TD2018 supplemented with 0.5% *w/w* choline (TD.180339), or 1.0% *w/w* choline (TD.180340) in the form of choline chloride purchased from Envigo (Indianapolis, IN). Mice (2–3 animals per cage) were kept on diets for 16 weeks. Blood was collected with EDTA at 0, 4, 8, 12 and 16 weeks of treatment through the retro-orbital plexus using isoflurane anaesthesia (3%) and plasma isolated by centrifugation. Mice were sacrificed, and tissues were removed for analysis. All animals were monitored and weighed weekly to ensure the health of the animals over the course of the study.

### 2.2. Plasma Analysis of Trimethylamine N-Oxide

Analysis of TMAO in plasma was performed at Touchstone Biosciences using a liquid chromatography-mass spectrometry (LC-MS/MS) method essentially performed as communicated by Koeth et al. [1]. A brief summarization follows. A volume of 10 µL of the plasma samples was pipetted into a 2 mL centrifuge tube, where 50 µL of HPLC-grade acetonitrile containing internal standard was then added. Samples were centrifuged (3000× *g* for 10 min), and supernatant was removed for analysis by LC-MS/MS. Calibration standards and quality controls in blank plasma were made by preparation of a 1 mg/mL TMAO stock solution and a subsequent series of working solutions in methanol: water (1:1, *v/v*), which were spiked into blank plasma to yield a series of calibration standard samples in the range of 10 ng/mL to 10 μg/mL and quality control samples at three concentration levels (30, 300 and 3000 ng/mL). The mixture was vortexed for 2 min, followed by centrifugation. All incurred plasma samples were treated identically to the calibration standards and quality control samples. HPLC-MS/MS analysis was performed using a Shimadzu HPLC and an AB/MDS Sciex MS/MS system. Elution was carried out on a Phenomenex Kinetex C18, 2.6 μm (4.6 × 50 mm). All analyses were performed at a column temperature of 30 ± 1 °C with a mobile phase (A) 0.1% formic acid in water and (B) 0.1% formic acid in acetonitrile. The detection and quantification of TMAO levels (ppm) was attained by ESI-MS/MS operating in the positive ion mode.

### 2.3. Lipoprotein Plasma Profiling

Total cholesterol (TC), triglycerides (TG), phospholipids (PL) and free cholesterol (FC) in mouse plasma were determined using colorimetric kits from Wako Diagnostics (Richmond, VA, USA). Cholesterol ester was defined by subtraction of free cholesterol from total cholesterol. High density lipoprotein-cholesterol (HDL-C), low density lipoprotein-cholesterol (LDL-C) and very low density lipoprotein-cholesterol (VLDL-C) were measured by gel electrophoresis using SPIFE3000 and Cholesterol-Vis kit from Helena Laboratories (Beaumont, TX, USA). Because plasma was also used to verify expression of CETP, there was insufficient amounts of sample for each animal at baseline to measure lipoproteins or lipids.

### 2.4. HDL Inflammatory Index (HII)

Using a cell-free assay, HII was measured as the functional ability of HDL to inhibit or promote oxidation of oxLDL. The assay was performed as previously described [15], with a modification of the oxidation of LDL. LDL was isolated by ultracentrifugation and then dialyzed in phosphate-buffered saline (PBS). LDL was oxidized by incubating freshly prepared LDL solution that was uncapped at room temperature for 1–2 h. The oxLDL was diluted to a concentration of 100 µg/mL as previously described [15]. The organic phospholipid 2′,7′-dichlorodihydrofluorescein diacetate (DCF) was prepared as previously described by Navab et al. [16]. After polyethylene glycol precipitation of apo B, the HDL-containing supernatant was used in the assay. Oxidized LDL (final concentration: 1.4 µg/mL), DCF (final concentration: 2.9 µg/mL) and a fixed volume of apo B-depleted serum from animals (5 µL) were incubated with PBS to a final volume of 175 µL in individual wells of a 96-well flat-bottom polypropylene microtiter plate (Fisher Scientific, Pittsburgh, PA, USA). The plate was subsequently incubated at 37 °C in a microplate spectrophotometer (Spectra Max M2, Molecular Devices, Sunnyvale, CA, USA). Serial excitations at 485 nm were completed every 90 s, complemented by automated plate shaking. Fluorescence at emission wavelength of 525 nm and a cut-off of 515 nm was measured after 1 h of incubation. Samples were analysed in duplicate, and mean fluorescence recorded. HII was defined as: RFU (oxLDL + HDL)/RFU (oxLDL), where RFU (oxLDL + HDL) is the mean relative fluorescence units (RFU) in the oxLDL + HDL wells, and RFU (oxLDL) is the mean RFU in the oxLDL-only wells. To ensure uniformity of batch effects, a pooled human serum control was used to correct for inter-assay variation across batches.

### 2.5. Measurement of Plasma Myeloperoxidase and Oxidized Phospholipids

Plasma myeloperoxidase (MPO) and oxidized LDL (oxLDL) were measured at 16 weeks of treatment using commercial ELISA assays according to the manufacturer’s protocols. Plasma MPO ELISA was purchased from ICL, Inc. (Portland, OR, USA). Plasma oxLDL ELISA was from Elabscience Biotechnology Inc. (Houston, TX, USA).

### 2.6. Assessment of Atherosclerosis

After 16 weeks of diet supplementation, animals were euthanized, and the thoracic aorta was isolated, trimmed of excess fat and formalin-fixed for 48–72 h for *en face* morphometric analysis. In addition, a small segment of the ascending aorta with the heart still attached was reserved for aortic root analysis. For *en face* analysis, aortas were splayed out, pinned on a black matrix and stained with Sudan IV as described in [17]. Images were captured using a Nikon Digital Sight DS-Fi1 camera connected to an SMZ45T stereoscope (Nikon, Inc., Melville, NY, USA). Following staining, morphometric analysis was performed using NIS Elements software (Nikon, Inc., Melville, NY, USA) to determine the lesion area.

For aortic root measurement, the heart with approximately 2–3 mm of ascending aorta was cut away from the aortic arch. The apex of the heart was removed, and the remaining heart with the attached aortic segment was placed in Tissue-Tek OCT (optimal cutting temperature) embedding medium (Sakura Finetek USA, Inc., Torrance, CA, USA) and finally frozen in a dry ice/2-methylbutane bath. Ten µm thick sections were cut from the ascending aorta through the entire aortic sinus until the ventricular chamber was reached. Sections were stained with hematoxylin–eosin and imaged using a Nikon Digital Sight DS-Fi1 camera connected to an Eclipse 50i microscope. The lesion areas at 5 stepwise sections (60 µm apart) spanning the entire aortic root were then quantitated using NIS Elements software (Nikon, Inc., Melville, NY, USA).

### 2.7. Statistical Analysis

Normal data were analysed separately by means of a 1-factor ANOVA with group as the main effect. Post hoc Tukey’s honestly significant difference test (HSD) pairwise comparisons were conducted to identify statistical differences between the groups when a significant main effect was found. Means that do not share a common letter designation are presented as significantly different. The α was set to 0.05 for all statistical analyses, and *p* < 0.05 was considered statistically significant. Before comparison of the treatment groups by parametric analyses, the Ryan–Joiner normality test (similar to the Shapiro–Wilk test) was conducted. Most of the data were observed to be normal among the treatment groups for outcomes. Nonparametric Kruskal–Wallis tests were performed if data were not normal for all groups of a measure. Results are expressed as mean ± SEM unless indicated otherwise. One animal from the control group was removed from all analyses due to remarkable observations in growth and fragile tissues at necropsy. Other missing data were due to not having enough samples for analysis. All statistical analyses were performed with Minitab^®^ software v. 17.2.1 (Microsoft Corp, Redmond, WA, USA).

## 3. Results

### 3.1. In Vivo Plasma Levels of TMAO

**Figure 1:**
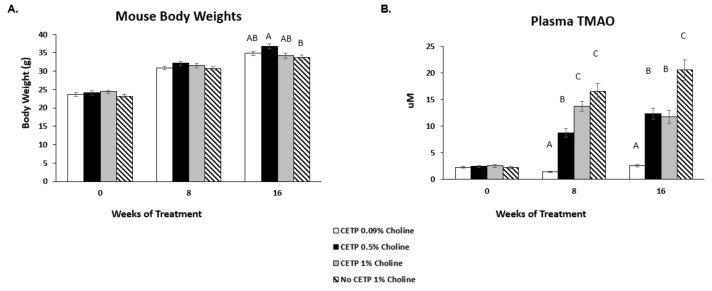
Mouse body weights and plasma TMAO levels in vivo. *Apoe^−/−^* mice were transfected with cholesterol ester transfer protein (CETP)-AAV at 7–8 weeks of age prior to choline supplementation. Animals were assigned to the indicated treatment groups at 9–10 weeks of age and fed diets for 16 weeks. (**A**) Mouse body weights for all treatments were evaluated to monitor overall health. (**B**) Plasma levels of trimethylamine N-oxide (TMAO) were measured at baseline, as well as 8 and 16 weeks after treatment. Data are presented as mean ± SEM (*n* = 10–12 mice/group). One-factor ANOVA test was used to analyse normally distributed data, and a Kruskal–Wallis test was performed for non-normally distributed data. Post hoc Tukey’s honestly significant difference test (HSD) pairwise comparisons were conducted to identify statistical differences between the treatment groups when a significant main effect was found. Means that do not share a letter are considered significantly different; *p* < 0.05.

To assess how choline feeding affects TMAO levels and atherosclerosis development, male *Apoe^−/−^* mice expressing CETP were assigned to 3 treatment groups fed 0.09%, 0.5% or 1% *w/w* choline. Plasma CETP expression levels were measured before the start of the choline supplementation to ensure consistent expression (14.3–15.5 µg/mL average per group; data not shown). Over 16 weeks of treatment, there were no significant differences in body weight between the CETP-transfected groups fed 0.09%, 0.5% or 1% choline. There was also no difference in body weight between the mice lacking CETP compared to those expressing CETP fed 1% choline (Figure 1A). Plasma TMAO levels were evaluated at baseline, as well as after 8 and 16 weeks on diets (Figure 1B). A significant dose-dependent increase in TMAO was observed at 8 weeks. However, at 16 weeks, plasma levels of TMAO in CETP mice were similar between the mid- and high-dose choline diet groups, with both significantly higher than those of the low-dose diet group. Interestingly, the plasma TMAO among mice fed the high-choline diet was significantly higher in the mice lacking CETP compared to those expressing CETP.

### 3.2. Lack of TMAO Effect on Aortic Lesions

**Figure 2:**
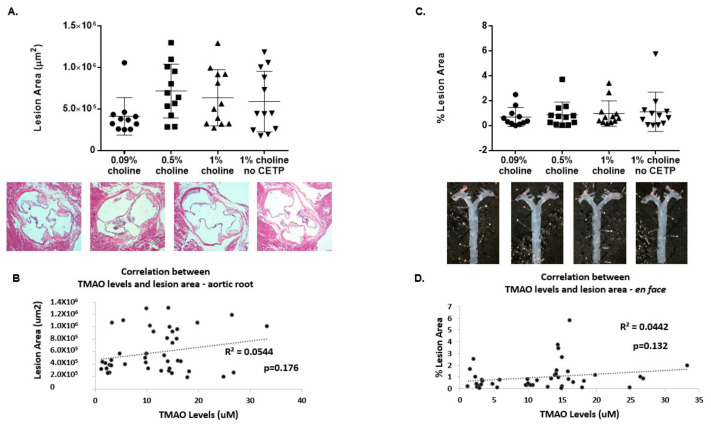
Effect of TMAO production on aortic lesions in vivo. *Apoe^−/−^* mice expressing CETP or controls (no CETP) were fed chow with supplementation of low, moderate and high choline for 16 weeks. Following 16 weeks of treatment, animals were sacrificed for aortic lesion assessments. (**A**) Sections (~10 μm) of the aortic root were cut, followed by hematoxylin–eosin staining (images below graph) and subsequent Nikon imaging. The total lesion area (μm^2^) across the aortic root for each treatment group was then plotted as the mean ± SEM from 11–12 animals per group. (**B**) Pearson correlation analysis was performed between aortic root lesion area and 16-week plasma TMAO concentrations for all groups (*n* = 43 mice). (**C**) Thoracic aortas were isolated, formalin-fixed and stained with Sudan IV for *en face* analysis (images under graph) using a Nikon computerized imaging system. The % lesion area in thoracic aortas for each treatment group was then expressed as the mean ± SEM from 11–12 animals/group. (**D**) Pearson correlation analysis was performed between % lesion area and 16-week plasma TMAO concentrations for all groups (*n* = 43 mice). One-factor ANOVA test was used to analyse normally distributed data, and a Kruskal–Wallis test was conducted for non-normally distributed data. Post hoc Tukey’s honestly significant difference test (HSD) pairwise comparisons were conducted to identify statistical differences between the treatment groups when a significant main effect was found. TMAO: trimethylamine N-oxide; CETP: cholesterol ester transfer protein.

At study terminus, animals were sacrificed for aortic lesion assessment at two common sites where atherosclerotic lesions develop: the aortic root and the thoracic aorta (including the aortic arch). Figure 2A shows the results of aortic root lesion analysis, including the total lesion area across the entire aortic sinus. Analysis of the aortic root lesion area (in µm^2^) showed no difference in lesion development between treatment groups. Subsequent analysis of lesion area as a function of TMAO concentrations for all animals did not result in a significant correlation, as seen in Figure 2B. Figure 2C shows results of *en face* morphometric analysis of the thoracic aorta from just above the heart to the bottom of the rib cage. Results indicate that there were no significant differences in lesion area among the different treatment groups in the thoracic aorta. Further analysis of lesion area in this region as a function of TMAO concentrations for all animals did not result in a significant correlation, as seen in Figure 2D. Even the high-dose choline-fed groups with and without CETP expression that showed significant differences in plasma TMAO levels showed no difference in lesion area at either site. Altogether, these results show that increased TMAO levels in response to increased choline intake do not affect atherosclerotic lesion development in *Apoe^−/−^* male mice expressing CETP.

### 3.3. Effects of Increased TMAO on Plasma Lipid Profile

**Figure 3:**
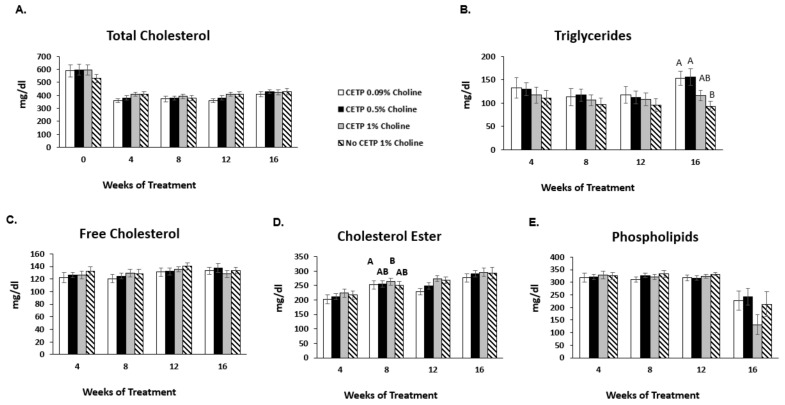
Effects of TMAO on plasma lipid levels in vivo. *Apoe^−/−^* mice expressing CETP or controls (no CETP) were fed chow with supplementation of low, moderate and high choline for 16 weeks. Blood from each animal was collected at baseline (for total cholesterol only) and at 4, 8, 12 and 16 weeks of treatment for plasma lipid analysis: (**A**) total cholesterol, (**B**) triglycerides, (**C**) free cholesterol, (**D**) cholesterol ester and (**E**) phospholipids were analysed. The results are expressed as the mean ± SEM from 11–12 animals/group. A one-factor ANOVA test was used to analyse normally distributed data, and a Kruskal–Wallis test was performed for non-normally distributed data. Post hoc Tukey’s honestly significant difference test (HSD) pairwise comparisons were conducted to identify statistical differences between the treatment groups when a significant main effect was found. Means that do not share a letter are considered significantly different; *p* < 0.05. TMAO: trimethylamine N-oxide; CETP: cholesterol ester transfer protein.

**Figure 4:**
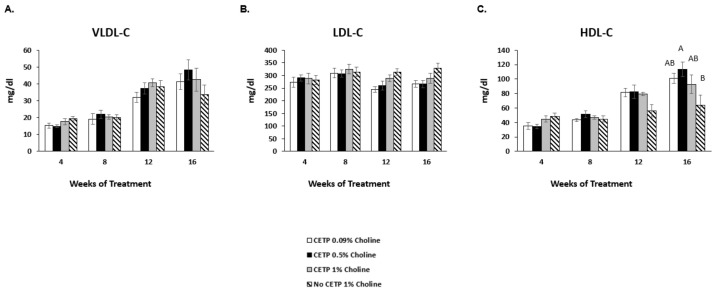
Effects of TMAO on lipoprotein particle distribution in vivo. *Apoe^−/−^* mice expressing CETP or controls (no CETP) were fed chow with supplementation of low, moderate and high choline for 16 wk. Blood was collected at 4, 8, 12 and 16 weeks of treatment. Plasma lipoproteins (**A**) VLDL-C, (**B**) LDL-C and (**C**) HDL-C were determined by gel electrophoresis using SPIFE3000 and a Cholesterol-Vis kit. The results are expressed as the mean ± SEM from 11–12 animals/group. A one-factor ANOVA test was used to analyse normally distributed data, and a Kruskal–Wallis test was performed for non-normally distributed data. Post hoc Tukey’s honestly significant difference test (HSD) pairwise comparisons were conducted to identify statistical differences between the treatment groups when a significant main effect was found. Means that do not share a letter are considered significantly different; *p* < 0.05. TMAO: trimethylamine N-oxide; CETP: cholesterol ester transfer protein; VLDL-C: very low density lipoprotein-cholesterol; LDL-C: low density lipoprotein-cholesterol; HDL-C: high density lipoprotein-cholesterol.

Total, free and esterified cholesterol, as well as phospholipids and triglycerides in plasma, were assessed at 4, 8, 12 and 16 weeks of choline supplementation. Only baseline total cholesterol was assessed due to insufficient amounts of plasma at that timepoint. Total cholesterol in all groups decreased at week 4 versus baseline due to the base diet change in all groups (Figure 3A). There were no differences in total cholesterol between treatment groups after 16 weeks of treatment. Additionally, no significant changes in free cholesterol, phospholipid or triglyceride levels across treatments were observed (Figure 3B,C,E). There was a small but significant increase in cholesterol ester in the high-choline-fed CETP-expressing mice versus the low-dose group at 12 weeks, but this effect did not persist at 16 weeks (Figure 3D). No significant differences were observed in plasma concentrations of the major lipoprotein classes (VLDL-C, LDL-C and HDL-C) between the CETP-transfected groups fed 0.09%, 0.5% or 1% choline or the mice lacking CETP compared to those expressing CETP fed 1% choline (Figure 4A–C).

### 3.4. Effects of TMAO on MPO, OxLDL and HDL Antioxidant Capacity

**Figure 5:**
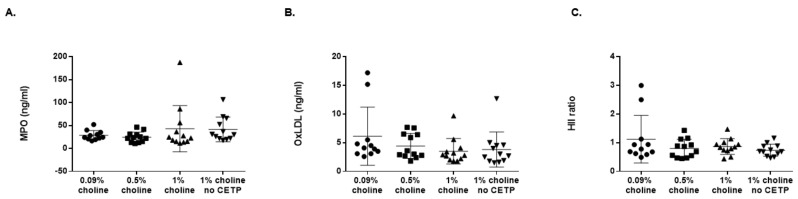
Choline supplementation did not affect plasma-oxidized lipids or HDL antioxidant capacity in vivo. *Apoe^−/−^* mice expressing cholesterol ester transfer protein (CETP) or controls (no CETP) were fed chow with supplementation of low, moderate and high choline for 16 wk. Concentrations in plasma of oxidized lipids (**A**) MPO and (**B**) oxLDL were measured. Additionally, (**C**) plasma HII (antioxidant capacity) was measured. Data are presented as mean ± SEM (*n* = 11–12 mice/group). A one-factor ANOVA test was used to analyse normally distributed data, and a Kruskal–Wallis test was performed for non-normally distributed data. Post hoc Tukey’s honestly significant difference test (HSD) pairwise comparisons were conducted to identify statistical differences between the treatment groups when a significant main effect was found. Means that do not share a letter are considered significantly different; *p* < 0.05. MPO: myeloperoxidase; oxLDL: oxidised low density lipoprotein; HII: HDL Inflammatory Index.

Plasma levels of MPO and oxLDL, as well as plasma HII, were measured after 16 weeks on the experimental diet (Figure 5A–C). No significant differences in either MPO or oxLDL plasma levels were observed. Similarly, HDL inflammatory index (a measure of antioxidant capacity) was not different between the treatment groups. These data suggest that these markers of inflammation and proatherogenicity were not altered by increasing TMAO levels due to an increase in choline in the diet.

## 4. Discussion

In the first metabolomics study linking choline and TMAO in the pathogenesis of CVD, Wang [2] suggested atherosclerosis in both the *Apoe^−/−^* female and male mice was associated with choline intake and plasma TMAO levels. After 16 weeks on either a normal chow, 0.5% or 1% choline, or 0.12% TMAO diet, aortic roots were examined and described as demonstrating a dose–response enhancement of atherosclerosis. We aimed to repeat this study in male *Apoe^−/−^* mice using the same treatment diets but in the presence of CETP. We included a 1% choline group without CETP as a comparison to the original study.

The aortic root lesion size in the male mice on normal chow and on a 0.5% choline diet in the Wang study was approximately 19,000 μm^2^ and 25,000 μm^2^ (*p* = 0.2), respectively, but lesion size, when fed 1% of the diet (10X the normal chow choline dose), which corresponds to a human dose of 10,000 mg choline per day, was approximately 37,000 μm^2^ (*p* = 0.045). After correcting the present study to average lesion area, for the control mice with CETP and 1% choline-fed mice with CETP, the average lesion area was 13,713 ± 7543 μm^2^ and 21,212 ± 11,308 μm^2^, respectively, similar to what Wang et al. reported [2]. Although the previous study just met statistical significance in the 1% choline supplemented group and given that the present study reported the same amount of lesion development as previously seen, we failed to see a statistically significant difference in lesion development in any of our groups supplemented with choline when compared to control.

In the present study, we found that TMAO concentrations in mice without CETP on a 1% choline diet were similar to those previous published (average concentration of ~20 μM). We also found a significant dose response for plasma TMAO after 8 weeks in the CETP-transfected mice, although at 16 weeks, TMAO levels were only significantly different between chow and 0.5% but not between 0.5% and 1% choline. An unexpected finding is that mice without CETP fed 1% choline had significantly higher TMAO levels than mice expressing CETP fed 0.5% and 1% choline (Figure 1B). Although in the current study, we achieved similar TMAO levels when compared to those reported by Wang et al., as well as a similar amount of aortic root lesion development, we saw no association of plasma TMAO concentration with lesion development in the presence or absence of CETP in male *Apoe^−/−^* mice when fed high doses of choline on a normal diet. The TMAO lesion area association that Wang et al. saw [2] represents 13 mice with TMAO concentrations greater than 20 μM. More than half of those mice had lesion areas similar to that of animals with TMAO concentrations near the lowest detectable limits.

Both Wang [2] and Koeth [1] cite the lack of change in various lipid and other inflammatory markers as supportive of a role for TMAO in the pathogenesis of atherosclerosis. In our study, we were also unable to demonstrate any difference in these markers between the treatment groups, as well as other changes in markers of inflammation, such as MPO, HDL inflammatory index or proatherogenic oxidized phospholipids.

To date, the results from Wang [2] have not been consistently duplicated when feeding high doses of choline or TMAO. Two additional independent groups have recently explored the connection between dietary choline in murine models of atherosclerosis [11,13]. Lindskog Jonsson [13] compared conventionally raised and germ-free *Apoe^−/−^* mice fed either a normal chow or a Western diet with or without choline supplement (chow plus 1.2% choline and Western diet plus 1% choline, respectively, which are equivalent to the highest dose used by Wang) [2]. After 12 weeks on the diets, the animals were sacrificed, and aortic roots were examined for lesion development. TMAO levels were elevated in the conventionally raised (CONV-R) choline cohort but not in the CONV-R control (no extra choline) chow group or either of the germ-free (GF) groups. This confirms that a 10-fold dose of choline raises TMAO levels only in CONV-R mice (which have a microbiota load of approximately 10^11^ vs. 100 for GF). Atherosclerotic lesions in the aortic roots were observed in both GF and CONV-R on conventional chow or Western diets, and if anything, the lesions were smaller in the CONV-R chow groups (with or without extra choline) and comparable in all Western diet groups. Moreover, there was no correlation between plasma TMAO concentration and lesion size development.

Aldana-Hernandez [11] examined the relationship in two atherogenic models. When no association between choline intake and atherosclerosis was found in the LDL receptor knockout model (*Ldl-r^−/−^*), it was assumed that the modest increase in TMAO was insufficient to induce cardiovascular changes, and higher doses of TMAO were given and feeding time was doubled. TMAO levels were elevated more in this group, but the mice still did not demonstrate a higher incidence of atherosclerotic plaque after 16 weeks. They repeated this in the *Apoe^−/−^* model and fed either normal chow (0.09% choline), high-dose (1% choline) or TMAO (0.12%). In contrast to the Wang [2] study, no increase in TMAO or atherosclerosis was observed after 12 weeks. Again, the working hypothesis was that there was insufficient time allowed for TMAO elevation or plaque development. Aldana-Hernandez [11] performed an additional feeding trial with the same dietary interventions, which was continued for 28 weeks, and although TMAO levels were shown to increase significantly, these mice did not demonstrate an increase in atherosclerotic plaque development as compared to control mice.

Lindskog Jonsson [13] found no difference in the effects of choline on atherosclerosis in male or female mice. Aldana-Hernandez [11] performed their experiments in male mice only. In an attempt to understand the differences between these two studies, the Getz and Reardon [18] posited that Wang [2] supplemented the diet starting at 4 weeks, compared to 8 weeks by Lindskog Jonsson [13], by which time the flavin-containing monooxygenase 3 protein enzyme (FMO3) is no longer expressed in male mice. However, this explanation however unlikely, as TMAO levels were elevated in both studies in much older mice, indicating the presence and adequacy of FMO3 in converting TMA to TMAO. In our study, we used only male *Apoe^−/−^* mice and administered the same amounts of choline as those described in the Wang [2] paper, and we were also able to demonstrate a comparable dose-response elevation of TMAO in mice when choline supplementation was started at 9–10 weeks of age.

Koeth [1] used a model identical to that of Wang [2] but fed the *Apoe^−/−^* mice carnitine (another trimethylamine compound found in meat and capable of being transformed by some microbial strains in the gastrointestinal tract into TMAO). This group concluded that dietary L-carnitine altered caecal microbial composition, increased TMA synthesis which resulted in TMAO production and increased atherosclerosis. Collins [12] replicated this experiment in male *Apoe^−/−^* mice transfected with CETP and failed to show any difference between low-dose and high-dose carnitine in the production of aortic atherogenic lesions or even a dose-response rise in plasma TMAO in response to a human-equivalent dose of 500 mg and 2000 mg per day.

In a separate in vitro experiment, Collins et al. exposed J774 mouse macrophages to a very high TMAO gradient and were not able to demonstrate foam cell development [12]—an essential component of the atherogenesis model. Not only was there no change in cholesterol uptake by macrophages, but no impact on cholesterol efflux from the macrophage could be detected, which is consistent with findings from Lindskog Jonsson [13]. TMAO elevations with administration of carnitine or choline were found by Collins [12], Lindskog Jonsson [13] and again in this current study. Although Aldana-Hernandez et al. [11] did not find elevated TMAO after feeding high-dose choline (1%), the *Apoe^−/−^* mice were also fed 0.12% TMAO, and this did not produce a higher incidence of atherosclerosis.

There are few groups independent of Wang and Koeth that have shown associations of plasma TMAO concentrations with atherosclerotic lesion size when administering choline or TMAO in the diet. Ding [19] fed 0.3% TMAO to 9-week-old male *Apoe^−/−^* mice for 8 weeks in a casein-based semi-purified diet. The diets used in the present study and the Wang and Koeth diets are certified diets made with plant-based protein, and they are not semi-purified. Ding et al. saw a significant increase in percent lesion area for TMAO-treated animals when compared to control animals. Chen [20] treated 8-week-old female *Apoe^−/−^* mice with a 1% choline diet for 4 months on a normal chow but gave limited information about the composition of the diets. Their study showed more advanced lesions. Thoracic aortas had approximately 30% lesion coverage in the control animals and upwards of 70% in the 1% choline-fed group. When compared to the present study for the same amount of time on chow, we saw less than 1.5% lesion coverage of the thoracic area in our control and choline-treated animals. In their study, aortic root lesions were also more advanced, more indicative to those seen in a Western-diet-fed animal when looking at the representative micrographs. The measures reported for aortic root lesion area were percent of total vessel, not total area, so there is further difficulty in interpreting and comparing results to those of the current study.

With these differences seen between study groups, the intestinal microbiome has been shown to be influenced by dietary components, such as protein source [21,22] and fat [23,24], and microbiome diversity (a measure of gut health) has been shown to change with dietary patterns [25]. These differences make it difficult to reproduce animal studies that distillate on metabolites generated by the gut microbiome that are not well controlled or if semi-purified diets are not used. Other factors, including animal background, source or procurement, housing conditions (single- or multi-housed, caging types, etc.) and facility containment levels also play a role in influencing the gut microbiome and atherosclerosis outcome [26,27].

## 5. Conclusions

In conclusion, our study failed to corroborate any association between TMAO and aortic atherosclerosis when feeding large doses of choline to non-CETP-expressing male *Apoe^−/−^* mice for 16 weeks. Enhancing reverse cholesterol transport in CETP-transfected *male Apoe^−/−^* mice also failed to show any difference in atherosclerosis despite a dose-responsive elevation of TMAO after 8 and 16 weeks. Taken together, these data from male *Apoe^−/−^* mice with or without expression of CETP do not support increased plasma TMAO as a causative agent of atherosclerosis.

## Data Availability

The datasets used and/or analyzed during the current study are available from the corresponding author upon reasonable request.
